# Wearable Use in an Observational Study Among Older Adults: Adherence, Feasibility, and Effects of Clinicodemographic Factors

**DOI:** 10.3389/fdgth.2022.884208

**Published:** 2022-06-10

**Authors:** Emily W. Paolillo, Shannon Y. Lee, Anna VandeBunte, Nina Djukic, Corrina Fonseca, Joel H. Kramer, Kaitlin B. Casaletto

**Affiliations:** ^1^Memory and Aging Center, Department of Neurology, Weill Institute for Neurosciences, University of California, San Francisco, San Francisco, CA, United States; ^2^Department of Psychology, Palo Alto University, Palo Alto, CA, United States

**Keywords:** digital health, Fitbit, aging, memory, physical activity, wearable adherence

## Abstract

**Introduction:**

Wearables have great potential to improve monitoring and delivery of physical activity interventions to older adults with downstream benefits to multisystem health and longevity; however, benefits obtained from wearables depend on their uptake and usage. Few studies have examined person-specific factors that relate to wearable adherence. We characterized adherence to using a wearable activity tracker for 30 days and examined associations between adherence and demographics, cognitive functioning, brain volumes, and technology familiarity among community-dwelling older adults.

**Methods:**

Participants were 175 older adults enrolled in the UCSF Longitudinal Brain Aging Study who were asked to wear a Fitbit^TM^ Flex 2 during waking hours for 30 days. Sixty two of these participants were also asked to sync their devices to the Fitbit smartphone app daily to collect minute-level data. We calculated adherence to wearing the Fitbit daily (i.e., proportion of days with valid activity data) and adherence to daily device syncing (i.e., proportion of days with minute-level activity data). Participants also completed a brain MRI and in-person cognitive testing measuring memory, executive functioning, and processing speed. Spearman correlations, Wilcoxon rank sum tests, and logistic regression tested relationships between wearable adherence and clinicodemographic factors.

**Results:**

Participants wore the Fitbits for an average of 95% of study days and were 85% adherent to the daily syncing protocol. Greater adherence to wearing the device was related to female sex. Greater adherence to daily device syncing was related to better memory, independent of demographic factors. Wearable adherence was not significantly related to age, education, executive functioning, processing speed, brain gray matter volumes, or self-reported familiarity with technology. Participants reported little-to-no difficulty using the wearable and all reported willingness to participate in another wearable study in the future.

**Conclusions:**

Older adults have overall high adherence to wearable use in the current study protocol. Person-specific factors, however, may represent potential barriers to equitable uptake of wearables for physical activity among older adults, including demographics and cognitive functioning. Future studies and clinical providers utilizing wearable activity trackers with older adults may benefit from implementation of reminders (e.g., texts, calls) for device use, particularly among men and individuals with memory impairment.

## Introduction

The population of older adults is growing worldwide (1). In conjunction, the prevalence of multimorbid geriatric health conditions is increasing faster than the rate at which effective healthcare resources for older adults are being implemented (2–4). There is a pressing need to identify targets for preventative medicine approaches to maintaining optimal health among this growing population. Physical activity is one modifiable behavioral factor that has been strongly and consistently linked to better health across many domains, including cardiovascular health, physical frailty, mental health, and cognitive functioning (5, 6). In addition to the public health and economic benefits of reducing the burden of multimorbidity among older adults (7, 8), there are also clear individual benefits including improvement in quality of life and prolonged functional independence (9). Still, physical activity interventions in clinical settings remain underused (10) and even when patients are advised or encouraged to increase their physical activity by their healthcare providers (11, 12), there may be little long-term follow up.

Wearable devices are a potentially feasible, accessible, and effective way to bridge the gap between research and implementation with regard to physical activity as a preventative health measure. In fact, wrist-worn wearables for tracking physical activity are gaining popularity, even among older adults. Recent estimates suggest that 17% of U.S. adults aged 50 or older already use activity watches/wearable trackers regularly (13). Smart activity watches passively track and transmit objective activity information to an accessible yet secure cloud-based storage system, circumventing prior approaches reliant on self-report, which are often biased by recall errors, social desirability effects, or state-dependent bias (14, 15). In addition to collecting real-time objective data, wearables are also capable of delivering real-time individualized interventions to increase physical activity, including prompts to move when the device detects lack of activity (16). Many observational studies and interventions have already been conducted in several pediatric and adult populations (17, 18); however, less is known about the best practices for using wearables in research studies or clinical interventions with older adults.

Several studies support the validity of wearables for measuring physical activity in older adults and willingness to use these devices. A wide range of devices have been validated as accurate measures of activity, including both research-grade and commercially available wrist-worn devices (19, 20). Although no studies to date have examined factors that relate to wearable adherence among older adults in the context of a study or intervention, prior work has examined factors that relate to naturalistic wearable use. For example, Kononova et al. (21) examined factors that facilitate real-world wearable use among older adults, with long-term users being strongly motivated by social support and collaboration, while short-term users seemed most focused on competitive desires to increase physical activity. Older adult perceptions and real-world uses of activity trackers have also been well-characterized, with studies showing overall high levels of acceptability (22); however, acceptability and subsequent use is still highly dependent on a number of factors including cost, privacy, personal motivation, understanding device purpose, and ease of use (22–26). While studies thus far have demonstrated that many older adults are able to engage with wearable devices, there appear to be many device-specific qualities and subjective perceptions about wearables that affect their naturalistic uptake. Furthermore, cognitive changes, including declines in processing speed, memory, and executive functioning occur with age, highlighting the need to consider how cognitive and brain health relate to wearable adherence (e.g., forgetting to wear the device due to memory problems) in this population. Given the need for better implementation strategies for preventative healthcare among the growing population of older adults, it is imperative to examine person-specific factors that might be barriers to wearable use in the context of a study or intervention.

Thus, the primary aims of this study are to: (1) characterize engagement with wearables for physical activity among older adults using data from an observational exercise study; (2) examine associations between adherence to wearable usage and demographics, cognitive functioning, brain volumes, and self-reported familiarity with technology; and (3) characterize feedback from a post-study questionnaire. We hypothesized that better adherence to wearable usage will be related to younger age, better cognitive functioning, larger brain volumes, and greater familiarity with technology.

## Materials and Methods

### Participants

This study cohort included 175 English-speaking older adults aged 55 years and older who were recruited from the UCSF Longitudinal Brain Aging Study at the UCSF Memory and Aging Center. This parent study totals 408 English-speaking participants (56% Female; age_mean =_ 76.5 years; education_mean_ = 17.4 years; 83% White, 3% Black/African American, 9% Asian/Pacific Islander, and 5% Other or Unknown Race). Inclusion criteria for the Longitudinal Brain Aging study enrollment consisted of being age 55 and older and having no history or current evidence of the following conditions: clinically significant stroke, acquired brain injuries, DSM-5 major psychiatric disorders, Multiple Sclerosis, Parkinson's Disease, major memory concerns or related diagnoses, active substance abuse, Diabetes Mellitus, Hepatitis C, Epilepsy, Blindness, Deafness, HIV, and Syphilis. The observational Fitbit study from which current study data were derived followed this same guidance, with no additional exclusion criteria. This study was approved by the UCSF Institutional Review Board. All subjects provided written, informed consent to voluntary research participation.

### Procedure

Participants were scheduled for a Longitudinal Brain Aging baseline or annual follow-up research visit, which took place in-person at the UCSF Memory and Aging Center. Participants represent community-dwelling functionally intact older adults living in the Bay Area. These comprehensive visits included cognitive testing, neuroimaging, and questionnaire completion. Preceding or following their standard visit, all subjects were also invited to participate in the observational Fitbit study on an opt-in basis. This study was described as an optional add-on to the primary longitudinal program and aimed to investigate the link between lifestyle factors (i.e., physical activity) and brain health. At the Fitbit study appointment, participants were asked to wear an actigraphy watch (Fitbit™ Flex 2 model) for 30 continuous days on the non-dominant wrist during all waking hours, including both active and sedentary time. They were instructed to charge the device every night and resume wearing it the following morning. A subset of 62 participants who owned smartphones agreed to download the mobile Fitbit app and sync their Fitbit device to the app once per day. The other 113 participants were not expected to complete a daily sync; instead, all Fitbit data was synced to the app by a research coordinator after study completion. Study FAQ sheets were provided to each participant and contained trouble-shooting and syncing details. Activity logs were also distributed as means for participants to record any deviations from the study protocol, such as forgetting to wear, sync, or charge the device on a given day. Research coordinators emphasized the observational nature of the study, and they encouraged participants to go about their daily activities as they usually would. To further minimize self-monitoring effects on behavior, Fitbit activity feedback was reduced as much as possible. Feedback was inherently limited by the minimalist design of Flex 2 model, which does not feature a visual screen display. In addition, all in-app activity tracking tiles were removed and all exercise-related Fitbit and mobile device goals and notifications were disabled. After 30 days of daily use, participants were contacted to return their Fitbit by mail using a provided, prepaid envelope. Interested participants were able to request post-completion summaries of their physical activity metrics. Collected physical activity data was then linked to all relevant standard visit measures captured on the same visit day or within 500 days of the Fitbit study start date. Thirty eight participants who completed Fitbit did not have cognitive testing or neuroimaging completed at their standard visit within this timeframe.

### Fitbit Data Collection

Fitbit accounts were individually created for each participant through the mobile app, either on the subject's smartphone or on a research iPad to accommodate any subjects not using a personal cellular device for the study. Each participant was assigned a unique, de-identified username for app sign up, and each Fitbit was directly paired to the participant's respective app account via Bluetooth connection. Daily in-app Fitbit syncing required basic WIFI connection. Performing manual, daily syncs was optimal, as it allowed for minute-level presentation of physical activity data and in-depth analysis of step cadence for this subset of participants. For participants who did not sync every day, Fitbit stores daily aggregate metrics (e.g., daily total step counts and mileage). Upon device return, all Fitbits were charged and synced a final time to capture any aggregate-level data that was not previously uploaded to the app. All Fitbit accounts were then connected to Fitabase, a platform specifically tailored for wearable research data management. All de-identified participant Fitbit data were then exported from Fitabase, cleaned, and analyzed in R.

### Measures

#### Study Adherence

We measured study adherence in two ways. First, we measured each participant's daily adherence wearing the device, which was calculated as the proportion of study days with >100 steps recorded. This step count cutoff was used to identify days when participants likely did not wear the device for any part of the day, following previous study approaches (27). Among the subset of participants who were asked to sync their device to the smartphone app daily, we also calculated the proportion of study days for which any minute-level data was collected, as an indicator of successful syncing events.

#### Cognitive Functioning

Participants completed a brief neuropsychological battery in person at their parent-study visit. Tests assessed three cognitive domains: memory, executive functioning, and processing speed. Sample based *z*-scores were calculated for individual tests and then averaged within each domain to create a composite *z*-score. The memory composite included the CVLT-II (total immediate recall, long delay free recall, and recognition discriminability) and Benson Figure Recall. The executive functioning composite included a modified version of the Trail Making Test requiring participants to serially alternate between numbers and days of the week (total time to complete), a Stroop interference task (number of correct items in 60 s), phonemic fluency (number of D words in 60 s), design fluency (D-KEFS Condition (1), and digit span backward (longest span). The processing speed composite included computerized visuospatial processing speed (reaction time) tasks previously described elsewhere (28). Higher scores indicate better performance for the memory and executive functioning domains, whereas lower scores indicate better performance for the processing speed domain (i.e., faster times).

#### Neuroimaging

Participants also completed magnetic resonance imaging (MRI) using a Siemens Prisma 3T scanner. Whole brain T1-weighted images were acquired sagittally using magnetization prepared rapid gradient-echo sequence (TR/TE/TI = 2,300/2.9/900 ms, α = 9°) with field of view of 160 × 240 × 256 mm and isotropic voxel resolution of 1 mm3. All T1-weighted images were inspected visually for quality before processing and images with excessive motion or artifact were excluded. The N3 algorithm was used to correct for magnetic field bias (29). SPM12's unified segmentation procedure was used for tissue segmentation (30). Diffeomorphic Anatomical Registration using Exponentiated Lie algebra (DARTEL) was used to create a study-specific template for warping individual participant T1-weighted images (31). Images were normalized and modulated within the study-specific template space using non-linear and rigid-body registration. Smoothing was performed using an 8-mm full width half maximum Gaussian kernel. Linear and non-linear transformations between DARTEL's space and International Consortium of Brain Mapping (ICBM) space were applied to facilitate registration with a brain parcellation atlas. Quantification of volumes was performed by transforming a standard parcellation atlas into ICBM space and summing all gray matter within parcellated regions of interest (32). Total intracranial volume (TIV) was calculated as the sum of gray matter, white matter, and cerebrospinal fluid. This study examined total gray matter volume and medial temporal lobe volume (i.e., bilateral entorhinal, parahippocampal, plus hippocampal volume) with TIV regressed out.

#### Technology Familiarity and Feedback Questionnaires

*Q*uestionnaires were available through the UCSF Qualtrics Web Survey platform and completed at the end of the visit on a research iPad or at home using distributed email survey links. The Technology Familiarity Questionnaire asked questions about participants' prior experiences using computers and technological devices, including if participants: (1) have ever used a wearable tracking device (i.e., Fitbit, Jawbone, Apple Watch), (2) own a “smartphone” (i.e., iPhone, Android), (3) experience difficulty when using computers, and (4) experience anxiety when using a computer, tablet, or smartphone. Questions 1 and 2 offered binary response options “Yes” or “No”. Questions 3 and 4 response options were based on a Likert scale from 1 (least affected by difficulty) to 5 (most affected by difficulty). After completion of the Fitbit study, participants completed the Post-Study Feedback Questionnaire, which asked questions about experience using the Fitbit for the duration of their participation. Participants were asked to rate overall: (1) satisfaction with participating, (2) degree of Fitbit interference in day-to-day life, (3) degree of Fitbit comfort over the course of 30 days, (4) degree of difficulty maintaining the Fitbit's charge and using the Fitbit wristband, and (5) degree of change to day-to-day activities caused by wearing the Fitbit. The response options followed a Likert scale from 1 (e.g., not at all interfering, not at all difficult, no change to day-to-day activities) to 5 (e.g., very satisfied, very comfortable to wear). The survey also asked whether subjects would participate in a future wearable devices study (Yes/No).

### Statistical Analyses

Descriptive statistics were used to characterize adherence to wearing the Fitbit daily, adherence to syncing the Fitbit daily, and responses to the post-study feedback questionnaire. To examine bivariate associations between adherence and demographic and clinical factors, Spearman correlations and Wilcoxon rank sum tests were used for continuous and categorical variables, respectively. These non-parametric statistical tests were used due to the skewed distribution of adherence rates. For any statistically significant (alpha = 0.05) bivariate relationship with clinical factors, follow up analyses were conducted to covary for demographics. Specifically, due to issues with skew, adherence was dichotomized (<90% adherence vs. ≧90% adherence) and logistic regression was used to examine the specified clinical factor as a predictor of adherence, covarying for age, sex, and education. All analyses were conducted using R, version 4.0.5.

## Results

Participant demographic and clinical characteristics are displayed in Table 1. Participants were 74 years old on average, majority female with more than college education on average, and mostly non-Hispanic White. This sample was also fairly active, with about 7,000 steps taken per day on average. Among the subset of participants with cognitive data (*n* = 137), a majority of participants were cognitively normal per consensus review. A majority of participants (70%) reported having prior experience with wearables. Participants also reported little-to-no difficulty or anxiety from using technology on average. Of note, the differences in tech-difficulty and tech-anxiety ratings between participants who were and were not asked to sync their device daily were not statistically significant (*p* > 0.05).

**Table 1:** Demographic and clinical characteristics (*N* = 175).

	**Mean (*SD*) or *N* (%)**
Age	73.65 (8.68)
Sex (female)	101 (58%)
Years of education	17.62 (1.93)
Race/Ethnicity	
White	151 (86%)
Black/African American	3 (2%)
Asian	20 (11%)
Other	1 (1%)
Average daily steps	6,964 (3,760)
Cognitive status (cognitively normal)^a^	133 (97%)
Memory z-score^a^	0.03 (0.79)
Executive functioning z-score^a^	0.20 (0.66)
Processing speed score^a^	2.59 (1.58)
Smartphone ownership (yes)^b^	103 (85%)
Prior wearable experience (yes)^b^	85 (70%)
Difficulty with technology^b^	0.55 (0.72) [range = 1–4]
Anxiety from technology^b^	0.13 (0.36) [range = 1–3]

a *N = 137*.

b *N = 121*.

Among all 175 participants enrolled in the study, there was a high rate of daily adherence, with participants wearing the Fitbit on an average of 89% of study days (range = 0–100%; IQR = 91–100%). Four participants had 0% adherence. Study records indicate that they reported not wearing the Fitbit for various reasons (e.g., lost the device, interfered with their own personal activity watch). Among the subset of 62 participants who were asked to sync the Fitbit device to the smartphone app on a daily basis, adherence to the protocol was still high. On average, participants were about 85% adherent to the daily syncing protocol (range = 3–100%; IQR = 82–100%).

Among the entire sample of 175 participants, adherence to wearing the Fitbit was not significantly related to age (Spearman's rho = −0.100; *p* = 0.186) or years of education (Spearman's rho = −0.110; *p* = 0.146); however, adherence was related to sex (Wilcoxon rank sum = 3111.5; *p* = 0.044) such that women (mean adherence = 94%) were more adherent than men (mean adherence = 83%). Greater adherence to wearing the Fitbit showed a small effect with better memory performances, but did not reach statistical significance (Spearman's rho = 0.145; *p* = 0.090). Adherence to wearing the Fitbit was not strongly related to executive functioning (Spearman's rho = 0.033; *p* = 0.701) or processing speed (Spearman's rho = −0.125; *p* = 0.167). Adherence was not significantly related to TIV-adjusted brain volumes (total gray matter: Spearman's rho = 0.048, *p* = 0.653; medial temporal lobe: Spearman's rho = 0.086; *p* = 0.421). Adherence was also not strongly related to reported difficulty with technology (Spearman's rho = 0.087; *p* = 0.343), technology-related anxiety (Spearman's rho = −0.055; *p* = 0.548), smartphone ownership (Wilcoxon rank sum = 1014.5; *p* = 0.486), or prior experience using wearables (Wilcoxon rank sum = 1507.5; *p* = 0.891).

**Figure 1:**
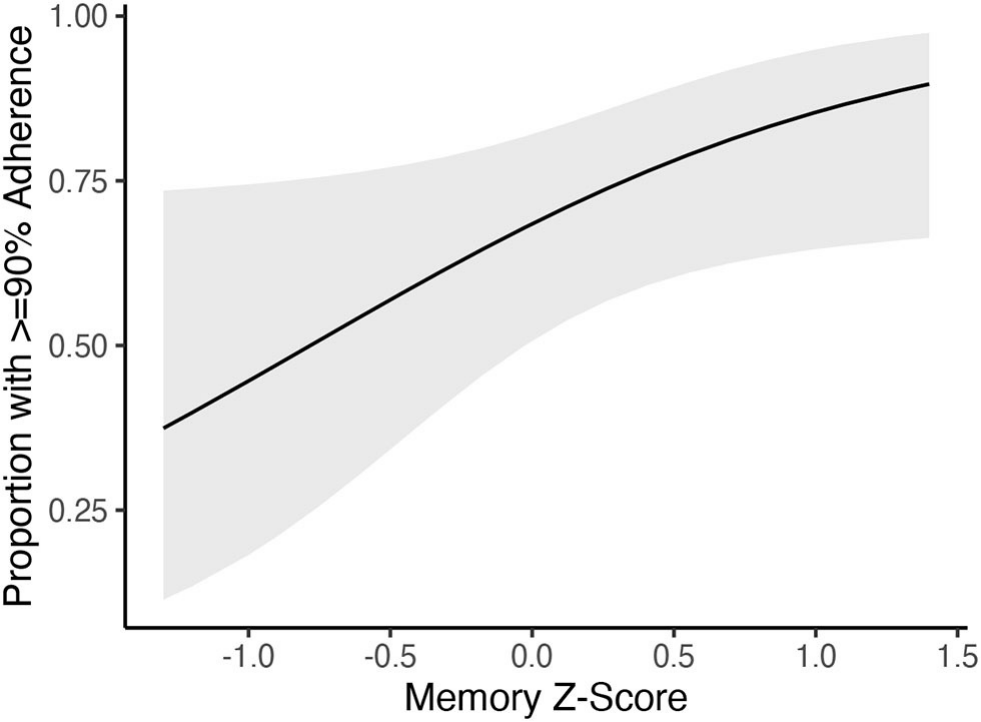
Better memory performance is associated with a greater likelihood of being at least 90% adherent to syncing the Fitbit daily.

Among the 62 participants with daily syncing, adherence to daily syncing was not significantly related to age (Spearman's rho = 0.162; *p* = 0.210), sex (Wilcoxon rank sum = 481; *p* = 0.939), or years of education (Spearman's rho = 0.086; *p* = 0.506). Greater adherence to daily syncing was significantly related to better memory (Spearman's rho = 0.356; *p* = 0.019). Follow-up logistic regression showed that memory remained a significant predictor of daily syncing adherence (OR = 2.69, 95%CI = 1.06–7.74, *p* = 0.046) even after covarying for age, sex, and education (Figure 1). Adherence to syncing the Fitbit was not statistically related to executive functioning (Spearman's rho = −0.019; *p* = 0.903) or processing speed (Spearman's rho = −0.076; *p* = 0.599). Adherence to syncing was not strongly related to brain volumes (total gray matter: Spearman's rho = 0.171, *p* = 0.349; medial temporal lobe: Spearman's rho = −0.022; *p* = 0.904). Adherence to syncing the Fitbit was also not strongly related to reported difficulty with technology (Spearman's rho = 0.040; *p* = 0.805), technology-related anxiety (Spearman's rho = 0.060; *p* = 0.714), or prior experience using wearables (Wilcoxon rank sum = 1507.5; *p* = 0.891).

**Figure 2:**
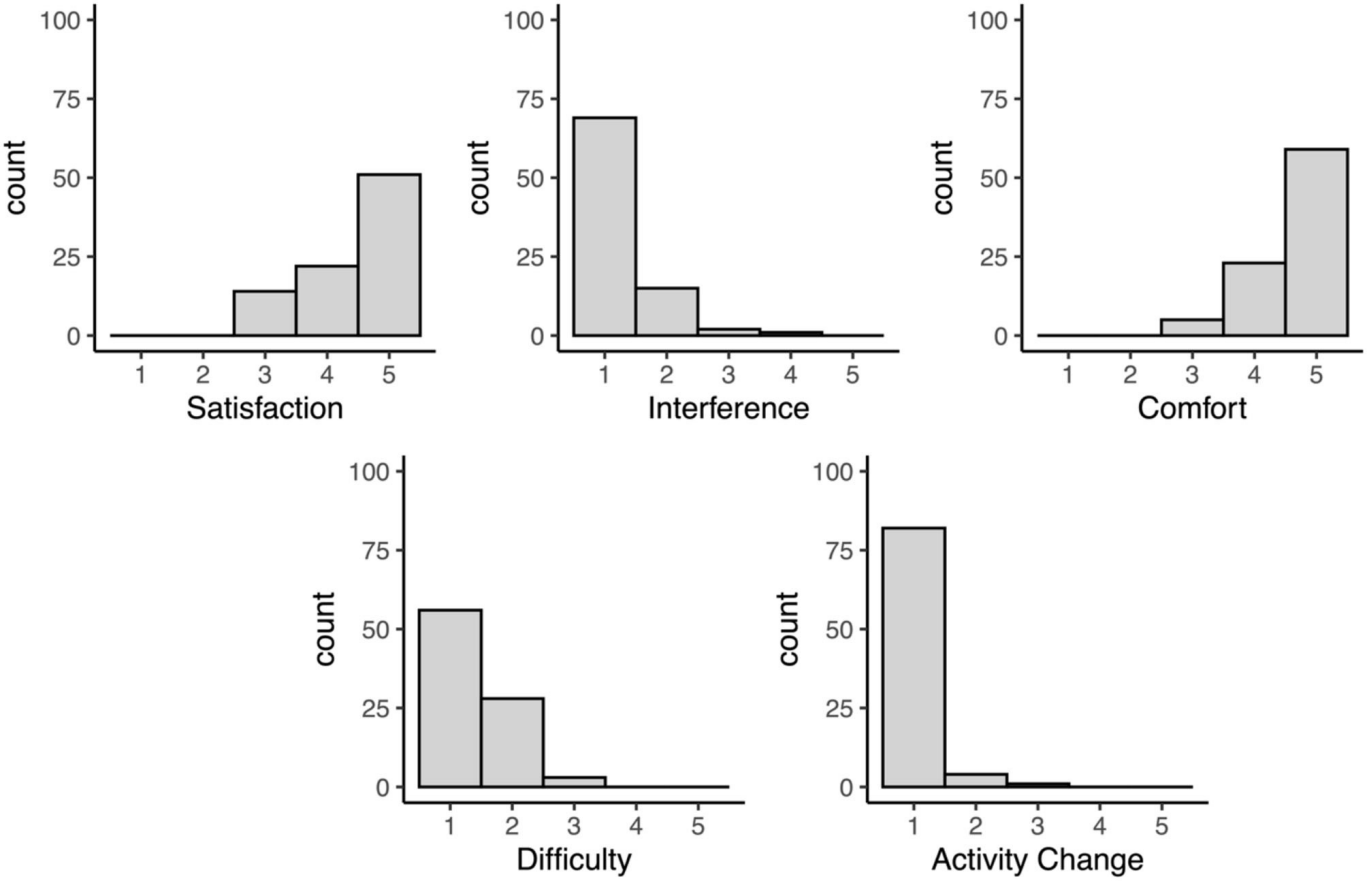
Distributions of responses to the study feedback questionnaire. All items are rated on a scale from 1 (not at all) to 5 (very much).

Finally, responses on the study feedback questionnaire were generally positive (Figure 2). Both participants who were and were not asked to sync their devices daily reported high satisfaction with their participation in the study (No daily syncing: mean = 4.42/5, *SD* = 0.78, range = 3–5; Daily syncing: mean = 4.48/5, *SD* = 0.69, range = 3–5) and high comfortability with wearing the Fitbit daily (No daily syncing: mean = 4.63/5, *SD* = 0.59, range = 3–5; Daily syncing: mean = 4.66/5, *SD* = 0.55, range = 3–5). On average, participants also reported that the Fitbit contributed little-to-no interference in their day-to-day life (No daily syncing: mean = 1.26/5, *SD* = 0.58, range = 1–4; Daily syncing: mean = 1.17/5, *SD* = 0.38, range = 1–2), low difficulty with charging the device and using the wristbands (No daily syncing: mean = 1.40/5, *SD* = 0.56, range = 1–3; Daily syncing: mean = 1.34/5, *SD* = 0.55, range = 1–3), and little-to-no change in their daily activities as a result of wearing the Fitbit (No daily syncing: mean = 1.05/5, *SD* = 0.29, range = 1–3; Daily syncing: mean = 1.10/5, *SD* = 0.31, range = 1–2). Additionally, all participants (100%) indicated that they would be willing to participate in another study using wearable devices in the future.

## Discussion

Given the increasing need to utilize effective behavioral interventions for prolonged health span among older adults, it is important to characterize the feasibility of wearable activity trackers and predictors of wearable use in a study context to inform future protocols and implementation procedures for clinical use. Adherence to wearable use is an important metric that can contribute to interpretation of observational and interventional exercise study effects, yet are often unreported (33). Our findings strongly support the use of wearable activity trackers for studies with older adults, including high overall adherence and satisfaction using the Fitbit in a way that was consistent with our study protocol. Results also suggest that sex and memory functioning may be important predictors of wearable adherence. The latter may be particularly relevant when individuals are required to manually sync devices with a smartphone app on a regular basis. Importantly, all participants indicated that they would be willing to use a wearable activity tracker again in another study the future.

Our findings showing high adherence rates for daily wearable use are generally consistent with previous studies in older adults, which have reported daily wearable use on as many as 98% of study days on average (34). Interestingly, we also found that female sex was associated with higher adherence to wearing the device daily. Although few previous studies have examined factors that predict wearable use among older adults, this sex-specific finding is somewhat consistent with at least one other report to our knowledge. Li and colleagues (35) found that women were more likely to be long-term (>6 months) wearable users in naturalistic everyday life than men. Such demographic factors are important to consider to ensure equity in the uptake of beneficial interventions using wearables.

We also identified a novel association between worse memory functioning and poorer adherence to device syncing in a cohort of otherwise functionally intact older adults. No prior studies to our knowledge have examined adherence to daily syncing, which allows for higher resolution data (e.g., steps per minute) to be collected. There was also some cognitive specificity, such that no strong associations between wearable adherence and executive functioning or processing speed were detected. The associations with memory are particularly notable given the high functioning status of our participants and raises potential concerns for wearable use among clinical, cognitively impaired samples. Our results suggest that studies using wearables in cognitively impaired populations may consider implementing daily reminders for use and manual syncing to minimize missing data. Other studies appear to have implemented successful reminder strategies among older adult participant samples, including phone calls or text messages. For example, one physical activity intervention study among older adults with cognitive impairment utilized reminders based on real-time data collection such that reminder calls were provided when no data was transferred to the cloud-based system for 3 consecutive days (36). Conversely, lower wearable adherence may even be used as a digital biomarker of memory status. Further research is needed to extend the work on passively-collected digital biomarkers of cognitive and everyday functioning in aging populations (37).

Finally, results from our study feedback questionnaire are very consistent with previous literature on acceptability of wearable use among older adults. Numerous studies have shown that older adults have high levels of acceptance and willingness to use wearable activity trackers (24, 38, 39). This is not unexpected given that a majority of U.S. older adults now own smartphones (40) and there is a slow but steady rise in uptake of digital health technology in general among this older population (41). This is promising for the integration of digital health technologies into research and clinical settings for improving our monitoring of modifiable lifestyle factors for maintaining optimal health into older adulthood.

## Limitations

This study was not without limitations. Our study involved a relatively small sample size. While there was a total of 175 subjects that participated in the study, only 62 of these subjects agreed to the continuous, manual syncing process by which they generated minute-level data and could be evaluated for daily syncing adherence. Our study also faced limitations of selection bias. Demographically, our study sample was largely limited to community-dwelling, cognitively healthy, mostly White, and highly educated older adults in the Bay Area. This study sample is demographically reflective of the broader UCSF Longitudinal Brain Aging Program from which participants were recruited and may not be generalizable to older adults in other geographic regions. This should be taken into account when considering the strong technological access and familiarity experiences dominantly reported by participants, and the overwhelmingly positive feedback reported about participating in the wearable study. Given that our study was completely observational, optional, and did not provide compensation, we expect that many of our older adult study volunteers may share a strong motivation to participate in research or other extracurricular activities and a particular interest in physical health and exercise. These characteristics reflect that this group may be more physically active and motivated to use wearables on average than the wider U.S. older adult population.

This possible selection bias also influences the generalizability of our study's findings. Further investigation, starting with the expansion of our wearables study to a broader range of older adults, is needed to better characterize and understand these feasibility, adherence, and memory-based relationships in the context of US older adults across different lifestyles and cognitive domains. The utility and feasibility of wearables in clinical older adult populations cannot be generalized by this study alone. Alongside many others, this study's sample highlights the crucial need for recruiting and including more diverse participant representation in our research across racial, socioeconomic, education, and cognitive diagnosis groups.

## Conclusions

Based on our comprehensive evaluation of study-specific wearable adherence, self-reported feedback, and capture of objective physical activity measures, wearables appear to be feasible and acceptable among community-dwelling older adults; however, consistent with previous studies, there are person-specific factors that likely affect regular daily use. Our findings support the continued use of wearable devices in studies with older adult populations to reliably track physical activity. Notably, adherence to wearable use should be monitored and reminders (e.g., texts, calls) may be particularly helpful in older adults at risk for memory difficulties. Technological upgrades to wearable devices now allow for automatic, Bluetooth-based data collection capabilities (i.e., without the need for manual syncing by the participant). Ideally, studies can utilize these newer wearable models and have devices seamlessly sync to a smartphone or other cloud-based system, which would eliminate participant syncing inconsistencies or errors almost entirely. These recommendations would help to streamline the data collection process and facilitate frequent, consistent device management on the participant side.

## Data Availability Statement

The raw data supporting the conclusions of this article will be made available by the authors, without undue reservation.

## Ethics Statement

The studies involving human participants were reviewed and approved by UCSF Human Research Protection Program (HRPP). The patients/participants provided their written informed consent to participate in this study.

## Author Contributions

EP contributed to study design, data analysis, interpretation, and manuscript writing and revision. SL contributed to data collection, study design, data analysis, interpretation, and manuscript writing. AV contributed to data interpretation and revision of manuscript for intellectual content. ND and CF contributed to data collection and revision of manuscript for intellectual content. JK contributed to study design, interpretation, and revision of manuscript for intellectual content. KC contributed to study design, data analysis, interpretation, and revision of manuscript for intellectual content. All authors contributed to the article and approved the submitted version.

## Funding

This study was supported by NIH-NIA grants K23AG058752 (PI: KC), R01AG072475 (PI: KC), 1R01AG032289 (PI: JK), R01AG048234 (PI: JK), and UCSF ADRC P30 AG062422 (PI: Miller). This work was also supported by Larry L. Hillblom Network Grant (PI: JK; 2014-A-004-NET) and the Alzheimer's Association (PI: KC; AARG-20-683875).

## Conflict of Interest

The authors declare that the research was conducted in the absence of any commercial or financial relationships that could be construed as a potential conflict of interest. The handling editor RM declared a past collaboration with the EP.

## Publisher's Note

All claims expressed in this article are solely those of the authors and do not necessarily represent those of their affiliated organizations, or those of the publisher, the editors and the reviewers. Any product that may be evaluated in this article, or claim that may be made by its manufacturer, is not guaranteed or endorsed by the publisher.
